# Impact of Pressure on Arsenic Released from Pore Water in Clayey Sediment

**DOI:** 10.3390/toxics10120738

**Published:** 2022-11-29

**Authors:** Cong Xiao, Yuzhu Chen, Teng Ma, Wen Xiong

**Affiliations:** 1 School of Civil Engineering, Architecture and Enivironment, Hubei University of Technology, Wuhan 430068, China; 2 School of Environmental Studies, China University of Geosciences, Wuhan 430074, China; 3 Cooperative Innovation Center of Industrial Fermentation (Ministry of Education & Hubei Province), Hubei University of Technology, Wuhan 430068, China

**Keywords:** arsenic, clayey sediment, compaction rate, compaction pattern, groundwater extraction

## Abstract

Overpumping can cause arsenic to be released from the pore water in clayey aquitards into aquifers. The amount of water pumped during groundwater exploitation may change over time, leading to different soil-compaction rates or patterns. However, the impact of pressure on the release of arsenic during the compaction of a clayey aquitard is poorly understood. We performed a laboratory-compaction experiment using clayey sediment to identify the effects of compaction rates and patterns on arsenic release by analyzing the chemical characteristics and arsenic species present in pore water samples collected at different stages of the compaction experiment. A rapid (PV increased linearly) and a slow (PV increased exponentially) water-release patterns were recognized according to the compaction rate. We observed that arsenic concentrations in the slow pattern (6.7 to 36.4 μg/L) were considerably higher than those in the rapid pattern (7.6 to 16.1 μg/L). Furthermore, concentrations were the highest in the accelerated compaction pattern (16.8 to 47.4 μg/L), followed by those in the constant and decelerated patterns (4.3 to 14.4 μg/L). Overall, compaction rate and pattern did not alter the arsenic-release mechanism; however, they did alter the moisture content of the sediment at each stage, which indirectly led to differences in the released arsenic concentrations. These results suggest that pumping rates and patterns must be considered to prevent arsenic contamination in groundwater-extraction scenarios.

## 1. Introduction

High concentrations of arsenic (As) in groundwater pose a threat to human health worldwide, and this threat is especially severe in Asia and the Americas [1,2,3,4,5]. Most people in these areas obtain drinking water from shallow tube wells, which can become contaminated with geogenic As. Deep aquifers generally have lower As concentrations than shallow aquifers [6,7,8]. Therefore, to reduce As exposure, many deeper community wells have been installed in recent years in regions where shallow aquifers are contaminated with As [9,10,11]. However, groundwater exploitation has increased since deeper low-As wells are in high demand [12,13,14,15]. Previous studies have found that groundwater overpumping has caused As concentrations to increase continually in deeper aquifers [6,10,16]. However, the specific mechanism of arsenic enrichment in deep aquifers under the influence of groundwater exploitation is still poorly understood.

It is generally believed that the clayey aquitards overlying deep aquifers can prevent arsenic pollution of shallow groundwater from entering deep aquifers [17,18]. However, the sediment in clayey aquitards is likely to be richer in dissolved organic carbon, microorganisms, heavy metals, and other contaminants than aquifer sediments [19,20,21,22]. Previous studies in recent years have shown that the As concentrations in deep low-As aquifers can increase as a result of As released by the compaction of less-permeable, clayey-aquitard sediment in response to groundwater overpumping [23,24,25]. Moreover, our research group has previously determined the biogeochemical reactions that occur during As release from clayey sediment via laboratory-compaction simulations [26] Statistics and simulation analyses of groundwater samples in the field found that the release of As from the pore water of clayey sediment is mainly mediated by pressure, which, in turn, is partly controlled by the magnitude of groundwater exploitation [27,28]. In addition, the volume of groundwater that is pumped from a specific exploitation area may change over time [15,29], leading to changing land-subsidence rates or producing different land-subsidence patterns (For example, the land-subsidence rate increases first and then decreases, rather than keeping a constant subsidence rate all the time). These differing land-subsidence patterns may affect the As released from clayey sediment during compaction. However, this process is difficult to observe in field studies. In addition, our team’s previous studies only analyzed the mechanism of arsenic release in clayey sediments at a specific land-subsidence rate. However, we did not explore whether land-subsidence rate and land-subsidence pattern would affect the concentration and release mechanism of arsenic in pore water released by clayey sediments. Thus, better understanding of the release characteristics of arsenic in clayey aquitards under different land-subsidence rates and patterns is urgently needed.

This study collected typical arsenic-rich clayey sediments under a reducing environment in the Jianghan Plain of China. The difference in subsidence rate is mainly due to the difference in layer-compression rate. In a laboratory experiment, the sediment- compression rate can be controlled by changing the compaction rate, so as to simulate the change in land-subsidence rate. Clayey sediments were compacted at three compaction rates and three compaction patterns in a laboratory-compaction-simulation apparatus to identify the role of pressure in As release from compressible clayey aquitards under groundwate- overexploitation scenarios. The results of this study can be used to better understand As enrichment in groundwater-exploitation areas.

## 2. Materials and Methods

### 2.1. Sample Collection

Clayey-sediment columns were collected from a 3 m × 1 m low-lying paddy field (30°16′26.3″ N, 113°43′44.6″ E) located in the east of Xiantao City, Jianghan Plain, central China. The sediment columns were collected 1 m beneath the surface using a sediment corer (KC hand-operated sediment corer-50820, KC-Denmark, Silkeborg, Denmark) in December 2017. The length and diameter of the core were 30 cm and 12.5 cm, respectively. The subsurface-clayey- sediment color was black–gray and had a high water content and low permeability. The bulk density of the clayey sediments was 1.13 g·C^−3^, the gravimetric-moisture content was 61%, and the total organic-carbon content was 0.19%. The minerals of clayey sediments are mainly composed of quartz (49%), clay minerals (montmorillonite (15%), chlorite (10%), illite (15%)), feldspar (5%), amphibole (3%), and calcite (3%). After removing the cultivated soil within 1 m beneath the surface, 5 parallel sedimentary columns were collected by the sediment corer and vacuum-sealed to minimize atmospheric exposure. All of the columns were stored at 4 °C until further analysis. 

### 2.2. Experimental Method

The clayey sediment was compacted using a low-pressure (<5 MPa), airtight-compression device [26]. The experimental device comprises a gas-pressure device, a compaction device, and a collection device, among which the compaction device is 40 cm tall and 12 cm in diameter. 

The possible oxidation parts of the clayey-sediment column (m = 3.41 kg) were scraped off before placing the column into the device to ensure unexposed sediment. The air in the device was expelled with argon before the piston was installed. The sediment column was also continuously supplied with argon as it was being placed into the device. After assembling the device, the sediment column was maintained in a closed state for 12 h prior to the compaction experiment. 

The compaction experiment consisted of two parts (Table 1). In the first part, three pressure rates (0.1 MPa/12 h, 0.1 MPa/24 h, and 0.1 MPa/48 h) were selected to study the effect of compaction rate on As release. The three pressure rates were divided into groups A, B, and C, and the pressure range of each group was 0–0.9 MPa. The pressure rates were achieved by adjusting the pressure regulator. Although the pressure rates herein differ from the actual sedimentation rate, the clayey aquitard’s compaction has been reflected to some extent [26]. In each group, 15 mL of pore water was collected from the column in anaerobic bottles for 10–20 min and preserved for analysis. The sampling intervals for groups A, B, and C were 12 h, 24 h, and 48 h, respectively, in the early stage. In the late stage, the sampling interval was 48 h. The sampling interval was set according to the volume of pore water required for analysis. 

In the second part of the compaction experiment, accelerated, constant, and decelerated pressurization experiments were performed to determine the effect of the compaction pattern of As release. The constant pressurization (groups A, B, and C) experiments used the same pressure rate throughout the compaction process, while the accelerated and decelerated pressurization experiments used early pressure rates that were lower and higher than the later pressure rates, respectively. For the accelerated pressurization experiment (group D), the pressure rate was 0.1 MPa/24 h from 0 to 0.4 MPa, and the sampling interval was 24 h. From 0.5 to 0.9 MPa, the pressure rate was increased to 0.1 MPa/12 h, and the sampling interval was 12 h. For the decelerated pressurization experiment (group E), the pressure rate was 0.1 MPa/12 h from 0 to 0.4 MPa, and the sampling interval was 12 h. From 0.5 to 0.9 MPa, the pressure rate was decreased to 0.1 MPa/24 h, and the sampling interval was 12 h. Approximately 15 mL of pore water was collected using anaerobic bottles for 10–20 min from groups D and E and preserved for further analysis.

### 2.3. Pore Water Analysis

Conductivity, pH, and Eh were measured using a HACH HQ40D multi-meter during the sampling period. Ferrous ions and ammonia concentrations were measured using a Hach 2800 portable spectrophotometer and Hach reagent kits (103769-CN ferrous ion and 2458200-CN ammonium reagents). The samples were immediately filtered through 0.45 μm membrane filters (MCE Syringe Filter) and divided into five aliquots for anion, cation, total-dissolved As, As(III), and dissolved organic carbon (DOC) analyses. Samples were acidified to pH < 2 using ultrapure HNO_3_ for cation analysis. Samples were acidified with concentrated HCl for total-dissolved As and As(III) analyses. For the DOC analysis, samples were preserved in 10 mL-brown-glass bottles and acidified with concentrated phosphoric acid (H_3_PO_4_) to pH 2.5.

The contents of major cations and anions of all pore-water samples were analyzed by Inductively Coupled Plasma Optical Emission Spectroscopy (ICP-OES) (PE avio 200, the detection limit of 0.01 mg/L) and Ion Chromatography (IC) (ThermoFisher Dionex Aquion with gradient elution; the detection limit is 0.01 mg/L), respectively. Total-dissolved As and As(III) were measured using hydride-generation-atomic-fluorescence spectrometry (HG-AFS; HG-AFS-930, Jitian, China), which has a detection limit of 0.01 μg/L. DOC was measured using a total-organic-carbon analyzer (Elementar Vario TOC cube) with a detection limit of 0.01 mg/L. These measurements were all performed at the China University of Geosciences.

## 3. Results

### 3.1. Pore Water Chemistry at Different Compaction Rates

After compaction, 738, 786, and 808 mL of pore water were released by groups A, B, and C, respectively. The cumulative amount of As released from the clayey sediment increased gradually during compaction, and the total amounts released from groups A, B, and C were 8.78, 19.02, and 16.88 µg, respectively. The analytical results of the water samples collected at different compaction rates are summarized in Table 2. All the pH showed a trend of decreasing and then increasing, and all the Eh values were, indicating that the experimental system exhibited a weakly to moderately alkaline-reducing environment during compaction.

The total As concentrations of the pore-water samples ranged from 7.6 to 16.1 μg/L in group A, from 11.1 to 36.4 μg/L in group B, and from 6.7 to 30.5 μg/L in group C, with average values of 11.7, 22.4, and 18.9 μg/L, respectively. The As(III) concentrations ranged from 2.0 to 7.3 μg/L in group A, from 3.3 to 22.0 μg/L in group B, and from 1.3 to 17.4 μg/L in group C, which accounted for 25.3–45.3%, 25.5–60.3%, and 19.4–57.0% of the total As, respectively. During the early compaction stage in groups A, B, and C, the NO_3_^−^ concentrations were high (7.64, 7.30, and 7.92 mg/L, respectively). The NO_3_^−^ concentrations decreased rapidly with increasing compaction time, and were low (2.46, 0.32, and 0.52 mg/L, respectively), when the experiment ended. In addition, the SO_4_^2−^ concentrations were low, and decreased gradually during compaction in all groups. Fe and Mn concentrations were relatively high in all groups (Table 2), and most of the values exceeded the limits of World Health Organization (WHO) guideline (0.3 and 0.5 mg/L, respectively). 

### 3.2. Pore Water Chemistry under Different Compaction Patterns

After compaction, groups D and E released 755 and 792 mL of pore water, respectively. The total As released by groups D and E were 24.52 and 7.54 µg, respectively. The pH and Eh characteristics of groups D and E are listed in Table 3.

The total pore-water-As concentrations ranged from 16.8 to 47.4 μg/L and from 4.3 to 14.4 μg/L in groups D and E, with averages of 32.3 and 10.0 μg/L, respectively. The As(III) concentrations ranged from 4.4 to 25.8 μg/L in group D and from 1.3 to 6.8 μg/L in group E, respectively, which accounted for 25.1–60.1% and 26.0–47.2% of the total As, respectively. 

During the early stage of compaction in groups D and E, the NO_3_^−^ concentrations were high (7.21 and 8.21 mg/L, respectively); subsequently, they decreased rapidly and were low (0.52 and 1.46 mg/L, respectively) at the end of the experiment. In addition, SO_4_^2−^ concentrations were low and decreased gradually during compaction in all groups. Fe and Mn concentrations were relatively high in each group (Table 3), and they exceeded WHO limits (0.3 and 0.5 mg/L, respectively) in most cases.

## 4. Discussion

### 4.1. Pore Water Releases at Different Compaction Rates

**Figure 1:**
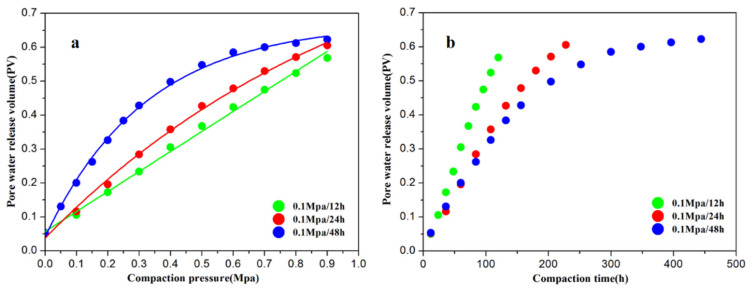
Variation of pore-water release with pressure and time at different compaction rates. (**a**) Relationships between the volume of pore water released from sediment and compaction pressure during compaction; (**b**) Relationships between the volume of pore water released from sediment and compaction time during compaction.

The relationship between the amount of pore water released and pressure is shown in Figure 1a. When the compaction rate was 0.1 MPa/12 h (group A), the released pore- water volume exhibited a linearly increasing trend (R^2^ = 0.99). In contrast, the released pore water exhibited trends of exponential increase when the compaction rates were 0.1 MPa/24 h (group B) and 0.1 MPa/48 h (group C) (R^2^ = 0.99 and R^2^ = 0.99, respectively). In addition, the final amount of pore water released from the clayey sediment in group C was slightly higher (22 mL) than in group B. The amount of pore water released over time is plotted in Figure 1b. Group A released pore water at a linear and constant rate. However, groups B and C released pore water at exponentially increasing rates, which decreased gradually with time. In addition, the rates at which pore water was released from groups B and C were similar prior to 100 h; however, group B’s release rate was greater than that of group C after 100 h. 

Figure 1 shows that the trends in the amount of water released from the clayey sediment differed under the three compaction rates. The amount of pore water released increased with decreasing compaction rate, and the total amount of pore water released remained constant when the compaction rate was less than a specific value. For groups A, B, and C, the fitting equation for the relationship between compaction rate and amount of pore water released was y = 8.005 × 10^2^–7.344 × 10^2^e^−0.2x^ (R^2^ = 0.99). When the compaction rate was less than 0.1 MPa/83 h, the total amount of pore water released was 800.5 mL. This value remained constant, mainly because bound water is difficult to release, and sediment porosity remains unchanged after being reduced to a certain extent by compaction [19,30,31]. The total amount of pore water released from group C was greater than 800 mL, as there was a deviation between the theoretical and actual values. 

Figure 1 shows that higher compaction rates produced higher pore-water release rates over the same period. However, at the same pressure, higher pore-water release rates had shorter release times, resulting in lower total amounts of released pore water. These results indicate that pore-water release patterns might differ at the three compaction rates. Owing to the high compaction rate of group A, the clayey sediment was subjected to high pressure over a short period, resulting in large porosity losses and rapid pore-water release from the sediment. In group A, the pore-water content decreased rapidly, and the rate of pore-water release was high and remained constant; thus, this trend of pore-water release with a nearly linear increase of pore-water volume was called the rapid-water-release pattern. Groups B and C were defined as the slow-water- release pattern, in which the rate of pore-water release decreased gradually, and the trend of pore-water release was a nearly exponential increase of pore-water volume over time throughout compaction. The compaction rate had little effect on the rate of pore-water release during the initial portion of the slow-water-release pattern (volume < 0.3), while a higher compaction rate during the final portion (volume > 0.3) produced a higher rate. During the initial portion of the slow-water-release pattern, the porosity of the clayey sediment was high, and the pore water could be released under a small compaction rate (assumed to be the critical compaction rate). According to the experimental results, the critical compaction rate was less than 0.1 MPa/48 h; thus, the rates of pore-water release in groups B and C had linear trends. During the final portion of the slow-water- release pattern, the constant release of pore water required a larger compaction rate; thus, the critical compaction rate during this period increased over time. Therefore, the rate of pore-water release from the sediment in groups B and C decreased gradually during this period. 

### 4.2. Effect of Compaction Rate on As Release 

**Figure 2:**
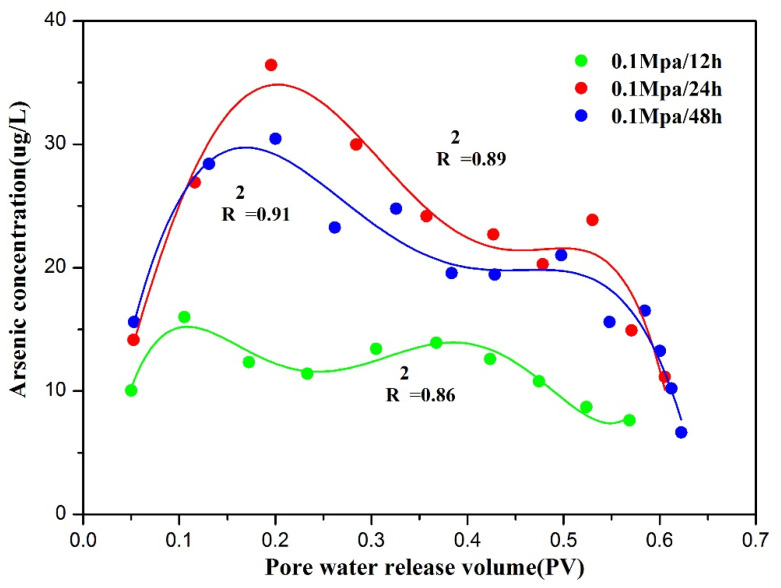
As concentrations in pore water released from sediments during compaction at different compaction rates.

According to Figure 2, the pore-water concentrations were essentially the same at all three compaction rates, exhibiting increasing–decreasing–increasing trends. However, there were significant differences in As concentrations between the rapid- (group A) and slow-water-release (group B and group C) patterns. When the compaction rates were 0.1 MPa/24 h and 0.1 MPa/48 h, the As concentrations of the pore water were similar, while those under a compaction rate of 0.1 MPa/12 h were substantially lower (Table S1). This difference indicates that the As-release mechanism differed between the rapid- and slow-water-release patterns. At higher compaction rates, less pore water was released, and the sediment-compression rate was lower, corresponding to a higher subsidence rate under actual groundwater overexploitation. Previous predictive models have indicated that As concentrations strongly correlate with the subsidence rate, and that higher subsidence rates increase the risk of As contamination [24,32], which is consistent with our experimental findings. 

**Figure 3:**
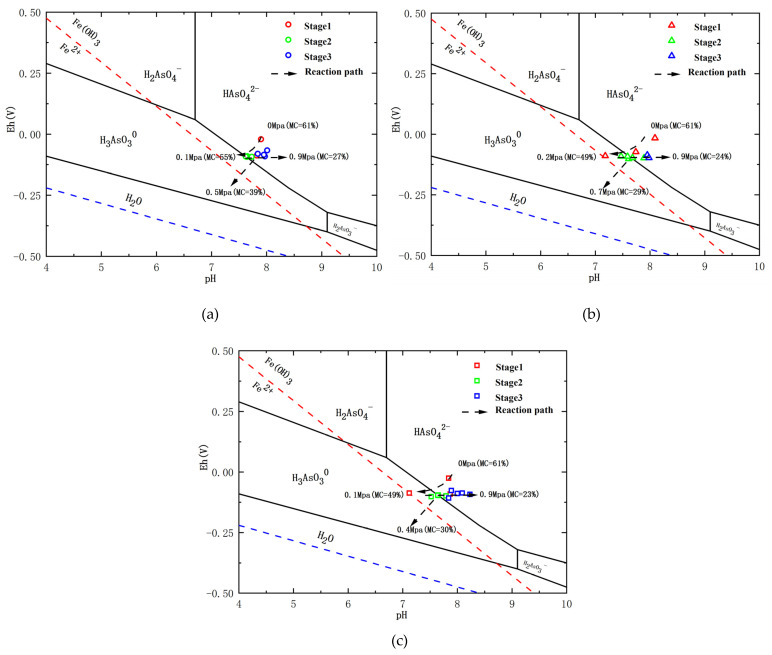
Eh–pH diagram of the As–Fe–H_2_O system in pore-water samples during compaction at different rates. The Eh and pH values of the pore-water samples are indicated using colored-open triangles for the different compaction stages. The solid-black lines indicate fields of As species. The red-dashed lines indicate fields of Fe species. The blue-dashed line delineates the boundary between H_2_ and H_2_O. (**a**) compaction rates of 0.1 MPa/12 h; (**b**) compaction rates of 0.1 MPa/24 h; (**c**) compaction rates of 0.1 MPa/48 h.

**Figure 4:**
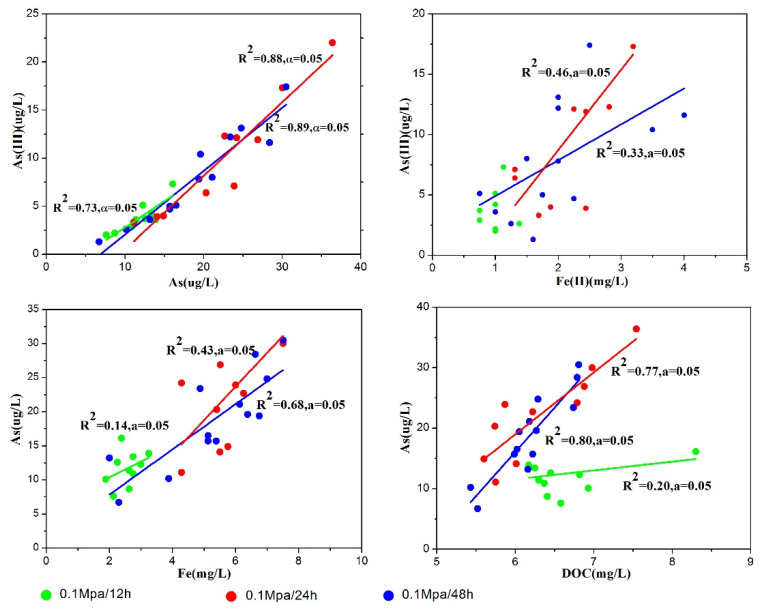
Relationships between As(III) and Fe^2+^ and between As and As(III)/Fe/DOC in pore water released from sediments during compaction at different rates. The red, blue and green lines respectively represent the corresponding linear relationships of the three groups.

**Figure 5:**
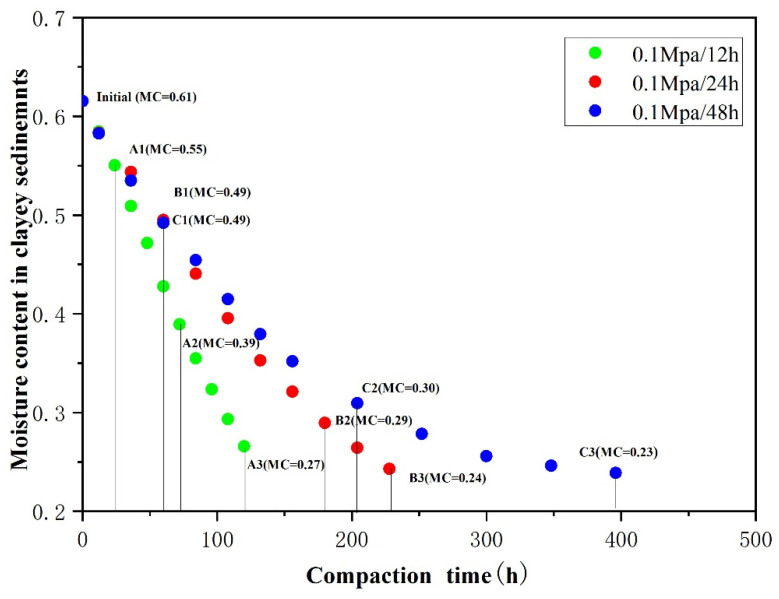
Moisture content of the clayey sediment during compaction at different compaction rates.

In our previous study [26], we evaluated that three stages can be recognized concerning the As-release mechanism during clay compaction at a rate of 0.1 MPa/24 h. The data obtained from the slow-water-release pattern exhibited the same As-release reaction path in an Eh–pH diagram of the As–Fe–H_2_O system during aquitard compaction (Figure 3). Variations in Eh during compaction led to the conversion of As species from As(V) to As(III) in stage 1, from As(III) to As(V) in stage 2, and no change from As(V) in stage 3. This result causes As release to change from a solid to an aqueous phase [33,34]. Moreover, when the compaction rates were 0.1 MPa/24 h and 0.1 MPa/48 h, As(III) was positively correlated with Fe(II) (R^2^ = 0.46 and R^2^ = 0.33, respectively), and the total As was positively associated with As(III), Fe, and DOC (Figure 4). These results indicate that the As-release mechanism in clayey sediments does not change over time in the slow-water-release pattern, and is not affected by the compaction rate. This release mechanism could be identified as the reductive dissolution of Fe oxides during stage 1 (moisture content > 49%), As desorption from Fe (hydr)oxides during stage 2 (moisture content of 49–30%), and As desorption from clay or carbonate minerals during stage 3 (moisture content < 30%) [26]. Moreover, when slow-pore-water release occurred in clayey sediments (compaction rates of 0.1 MPa/24 h and 0.1 MPa/48 h), the As concentrations during each stage were essentially the same, and the moisture content did not change (Figure 5). Hence, the effect of the compaction rate on As release was insignificant.

Although the As-release process of the rapid-water-release pattern can also be divided into the same three stages (Figure 3), the As concentrations and species differed from those of the slow-water-release pattern. Unlike the slow-water-release pattern, the rapid-water-release process resulted in low As concentrations, and the As species remained as As(V). The poor correlation between As(III) and Fe^2+^ further indicates the occurrence of a weak reductive dissolution of Fe oxides throughout the entire compaction process. In addition, poor correlations were observed between As and Fe, and between As and DOC in all stages (Figure 4), resulting in lower As concentrations and As(III)/As ratios for the rapid-water-release pattern, indicating that a weak reductive dissolution of iron oxides occurred [35,36]. Furthermore, compared with the slow-water-release pattern, higher moisture contents and shorter compaction times were observed for each stage in the rapid-water-release pattern (Figure 5). As was released before the pore water and clayey sediment reacted adequately during each stage, it resulted in lower As concentrations in the pore water [37,38]. According to the arsenic concentration and other hydrochemical data, the intensity of the reaction in each stage was significantly lower than that in the slow-water-release pattern.

In general, As concentrations of the pore water in the slow-water-release pattern were much higher than those in the rapid-water-release pattern during the same stage. The residual water amount in the clayey sediment during all stages of the slow-water- release pattern was also much lower than the rapid-water-release pattern (Figure 5), indicating that the compaction rate directly controls the sediment-moisture content [39], thereby affecting As released from the clayey sediment. In addition, the moisture content of the clayey sediment in the rapid-water-release pattern differed significantly at different compaction rates, while different compaction rates in the slow-water- release pattern had little effect on the sediment-water content. Thus, the compaction rate significantly affected As release in the rapid-water-release pattern, but had little effect on As release in the slow-water-release pattern.

### 4.3. Effect of Compaction Pattern on As Release

**Figure 6:**
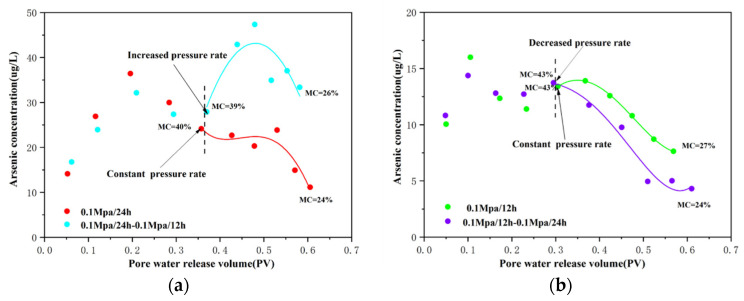
Arsenic concentration in pore water released from sediments under different compaction patterns. (**a**) Relationships between the volume of pore water released from sediment and arsenic concentration under the accelerated compaction patterns; The red and blue lines represent the trend of arsenic concentration in constant and accelerated compaction pattern, respectively; (**b**) Relationships between the volume of pore water released from sediment and arsenic concentration under the decelerated compaction patterns; The green and purple lines represent the trend of arsenic concentration in constant and accelerated compaction pattern, respectively.

The amounts of pore water released under the accelerated and decelerated compaction patterns were 755 and 792 mL, respectively, while those under the constant compaction pattern were 786 and 738 mL, respectively. The amounts of pore water released under the three compaction patterns were, in decreasing order: decelerated > constant > accelerated. The porosity loss of the decelerated compaction pattern was the largest, while that of the accelerated compaction pattern was the smallest. Larger porosity losses yield larger pore-water discharges; thus, the decelerated compaction pattern had the largest pore-water release. The As concentrations of the clayey sediments under the accelerated and decelerated compaction patterns are shown in Figure 6. The As concentrations of the pore water from the accelerated compaction pattern were higher than those from the constant compaction pattern. In comparison, pore-water As concentrations from the decelerated compaction pattern were lower than those from the constant compaction pattern. 

In the accelerated compaction pattern, when the compaction rate increased, the As(III)/As value of the pore water was less than 0.5, and the total Fe and Fe(II) concentrations were slightly higher than those during the same period in the constant compaction pattern (Table S2); however, the Fe(II)/Fe value was still less than 0.5. It indicates that the desorption reaction of As from the mineral surfaces in stage 3 still occurred [40,41,42]. When the compaction rate changed from 0.1 MPa/24 h to 0.1 MPa/12 h, rapid pore-water release occurred, and the amount of pore water released decreased, resulting in higher sediment-moisture contents (from 39% to 26%) during this process than under the constant compaction pattern (from 40% to 24%). At higher moisture contents, the sediment contributed As to the pore water via abiotic and biotic desorption [43]. Plus, with the reduced amount of pore water released, the higher moisture content allowed the sediment to react more fully with the pore water [44], resulting in a sudden increase in pore-water As concentrations at the end of stage 2 and in stage 3. As the main reactions during this process remained unchanged, the pore-water As concentrations increased overall. 

**Figure 7:**
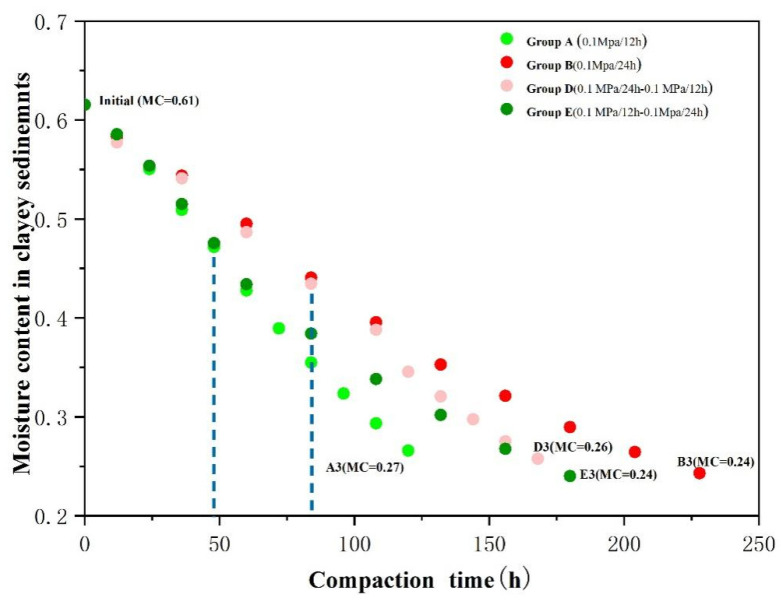
Sediment-moisture contents under different compaction patterns.

In the decelerated compaction pattern, when the compaction rate decreased, the variations in total As, As(III), total Fe, and Fe(II) concentrations of the pore water, with the amount of pore water released, were similar to those observed in the constant compaction pattern, indicating that the differences between the pore water released by these two patterns were small. After the compaction rate decreased, the sediment-moisture content in the decelerated compaction pattern (from 43% to 24%) was lower than in the constant compaction pattern (from 40% to 27%)(Figure 7). Low moisture contents indicate that the dissolved As concentrations decreased [43] and the amounts of pore water released increased, suggesting that the As concentrations of the pore water decreased.

### 4.4. Connections between Physical Processes and As Release

Owing to severe groundwater pollution and land subsidence that have occurred in recent years as a result of groundwater extraction, clay compaction has received attention in hydrology and hydrogeochemistry [1,45,46,47,48,49]. A few studies have shown that overpumping can cause As to be released from the pore water of clay aquitards into aquifers [2,6,10,24,25]. Clay aquitards have traditionally been considered non-point sources that release As at a constant rate during groundwater extraction [50]. This viewpoint ignores dynamic-pore-water-chemistry changes [25], including As concentrations, during clayey aquitard compaction. Although a quantitative model using the subsidence rate as a variable has indicated that As concentrations are correlated with groundwater extraction [10,24], the numerical simulation was limited to describing the interactions between physical compaction and As released as a result of clayey-aquitard compaction. In addition, dynamic- As concentrations with groundwater extraction is difficult to obtain in the field.

Using a laboratory-compaction simulation, we concentrated on the physical and chemical processes during clay compaction, establishing the connection between pressure rate, pressure pattern, and As concentrations in pore water. As discussed in the previous section, the variations in pore-water As concentrations and the variations in sediment- moisture contents under physical pressure are related. The variations in pore-water As concentrations depend on decreases in moisture content and increases in effective stress, both of which are caused by changes to the physical process (compaction rate and pattern). The physical processes and As release during clay compaction are related to sediment-moisture content.

## 5. Conclusions

In order to study the effects of compaction rates and patterns on arsenic release during clayey-sediments compaction, pore-water samples were collected at different stages of the laboratory-compaction experiment with different compaction rates and compaction patterns. Our experimental results indicate that rapid- and slow-water-release patterns from the clayey sediment can be identified according to the compaction rate. As release mechanism was the same in both patterns, but As concentrations in pore water in the slow pattern were significantly higher than those in the rapid pattern. As concentrations in pore water in the accelerated compaction pattern were higher than those in the constant compaction pattern. Furthermore, in the decelerated compaction pattern, As concentrations were lower than in the constant compaction pattern. The connection was established herein between compaction pressure and As concentrations resulting from As release from clayey aquitards. Our results indicate that compaction rate and pattern do not alter As-release mechanism; however, changes in the moisture content in the sediment indirectly lead to differing As concentrations released from clayey sediment. These results suggest that pumping rates and patterns must be considered to prevent As contamination in groundwater-extraction scenarios. In future research, the experimental findings can be combined with advanced field data to predict the impact of groundwater-extraction rates and patterns on arsenic contamination in deep aquifers.

## Figures and Tables

**Table 1:** Parameters of the three experimental groups in the compaction experiments.

Group	Initial Pressure	Final Pressure	Compaction Rate
A	0 MPa	0.9 MPa	0.1 MPa/12 h
B	0 MPa	0.9 MPa	0.1 MPa/24 h
C	0 MPa	0.9 MPa	0.1 MPa/48 h
D	0 MPa	0.9 MPa	0.1 MPa/24 h at 0–0.4 Mpa, 0.1 MPa/12 h at 0.5–0.9Mpa
E	0 MPa	0.9 MPa	0.1 MPa/12 h at 0–0.4 Mpa, 0.1 MPa/24 h at 0.5–0.9 Mpa

**Table 2:** Characteristics of pore-water samples collected under different compaction rates.

Sample	Eh	pH	EC	As	As(III)	DOC	Fe	Mn	NO_3_^−^	SO_4_^2−^	Fe^2+^	NH_4_-N
	mV		μs/cm	μg/L	μg/L	mg/L	mg/L	mg/L	mg/L	mg/L	mg/L	mg/L
A-12	−20.5	7.9	636	10.1	2.6	6.93	1.88	1.11	7.64	7.83	1.38	0.50
A-24	−87.5	7.82	666	16.1	7.3	8.30	2.38	1.31	7.47	6.50	1.13	1.00
A-36	−93.4	7.64	682	12.3	5.1	6.82	3.00	1.43	6.18	6.25	1.00	0.75
A-48	−90.6	7.65	682	11.4	3.6	6.30	2.63	1.52	6.24	5.78	1.00	0.88
A-60	−92.0	7.62	683	13.4	4.2	6.25	2.75	1.6	5.55	5.52	1.00	1.25
A-72	−92.7	7.73	676	13.9	3.6	6.17	3.25	1.62	5.88	5.87	1.00	1.50
A-84	−80.6	7.84	672	12.6	3.7	6.45	2.25	1.63	5.35	5.44	0.75	1.25
A-96	−91.3	7.97	673	10.9	2.9	6.37	2.75	1.67	4.93	5.00	0.75	1.00
A-108	−84.5	7.95	667	8.7	2.2	6.41	2.63	1.65	3.70	4.99	1.00	1.25
A-120	−66.3	8.01	615	7.6	2.0	6.58	2.13	1.64	2.46	4.87	1.00	1.25
B-12	−15.7	8.09	794	14.1	3.9	6.01	5.50	0.97	7.30	7.09	2.44	2.50
B-36	−73.2	7.74	832	26.9	11.9	6.88	5.51	1.05	5.86	6.11	2.44	1.75
B-60	−89.3	7.18	777	36.4	22.0	7.54	7.00	1.16	3.73	5.27	2.63	2.75
B-84	−91.1	7.47	851	30.0	17.3	6.98	7.50	1.15	2.61	5.54	3.19	2.25
B-108	−101.9	7.61	851	24.2	12.1	6.79	4.28	1.15	1.83	5.25	2.25	0.75
B-132	−91.6	7.59	844	22.7	12.3	6.22	6.25	1.13	1.66	5.20	2.81	1.25
B-156	−97.2	7.68	844	20.3	6.4	5.74	5.40	1.12	1.08	4.96	1.31	2.50
B-180	−97.9	7.89	840	23.9	7.1	5.87	6.00	1.11	0.57	3.56	1.31	2.00
B-204	−86.0	7.95	839	14.9	4.0	5.60	5.75	1.11	0.56	3.93	1.88	2.00
B-228	−97.4	7.98	842	11.1	3.3	5.75	4.28	1.09	0.32	3.03	1.69	1.75
C-12	−25.6	7.84	849	15.7	4.7	6.22	5.38	1.17	7.92	7.52	2.25	2.00
C-36	−91.6	7.87	860	28.4	11.6	6.79	6.63	1.14	6.48	6.16	4.00	1.25
C-60	−87.4	7.12	796	30.5	17.4	6.81	7.50	1.23	5.84	5.92	2.50	2.00
C-84	−102	7.52	843	23.4	12.2	6.74	4.88	1.23	2.48	5.23	2.00	1.75
C-108	−97.2	7.65	850	24.8	13.1	6.29	7.00	1.20	1.66	5.36	2.00	1.00
C-132	−95.8	7.65	835	19.6	10.4	6.27	6.38	1.18	0.65	5.29	3.50	2.00
C-156	−96.4	7.64	845	19.4	7.8	6.05	6.75	1.17	0.57	4.90	2.00	2.25
C-204	−98.9	7.79	842	21.1	8.0	6.18	6.13	1.16	0.55	3.94	1.50	1.25
C-252	−107.8	7.84	836	15.7	5.0	5.99	5.13	1.12	0.58	3.79	1.75	1.25
C-300	−86.8	8.09	834	16.5	5.1	6.02	5.13	0.55	0.60	3.89	0.75	1.50
C-348	−92.5	8.23	815	13.2	3.6	6.16	2.00	0.55	0.50	3.42	1.00	1.50
C-396	−89.0	8.00	762	10.2	2.6	5.43	3.88	0.44	0.58	3.23	1.25	1.50
C-444	−76.6	7.89	764	6.7	1.3	5.52	2.30	0.41	0.52	3.52	1.60	1.25

**Table 3:** Characteristics of pore-water samples under different compaction patterns.

Sample	Eh	pH	As	As(III)	DOC	Fe	Mn	NO_3_^−^	SO_4_^2−^	Fe^2+^	NH_4_-N
	mV		μg/L	μg/L	mg/L	mg/L	mg/L	mg/L	mg/L	mg/L	mg/L
D-12	−18.9	7.94	16.8	4.4	5.97	5.25	0.88	7.21	7.95	2.25	0.50
D-36	−59.9	7.74	23.9	11.5	6.79	5.75	0.88	5.67	6.22	2.00	0.75
D-60	−94.0	7.23	32.1	17.2	6.90	6.50	0.93	3.04	6.02	1.50	1.25
D-84	−108.9	7.58	27.4	15.3	5.85	5.25	1.00	2.24	5.81	1.50	1.25
D-108	−97.5	7.53	27.9	13.4	5.71	4.75	1.02	1.46	5.76	2.25	1.25
D-120	−95.0	7.61	42.9	25.8	4.49	4.00	0.78	0.57	5.79	2.50	1.25
D-132	−91.4	7.71	47.4	21.5	4.99	3.75	0.59	0.51	5.33	1.75	1.00
D-144	−96.6	7.75	34.9	11.0	3.32	5.75	1.1	0.54	4.51	1.50	1.00
D-156	−88.9	7.73	37.0	11.3	2.21	4.50	1.09	0.58	4.75	2.75	1.25
D-168	−90.5	7.77	33.4	8.4	1.71	5.50	1.08	0.52	3.23	2.00	1.00
E-12	−18.1	7.91	10.8	2.9	6.67	2.00	0.91	8.21	7.12	1.25	2.50
E-24	−62.3	7.83	14.4	6.8	8.92	2.00	0.46	7.88	7.07	0.75	1.75
E-36	−75.0	7.67	12.8	5.4	6.95	2.00	0.98	5.91	6.83	1.00	2.75
E-48	−73.9	7.70	12.7	4.6	6.90	2.63	1.05	5.45	6.98	0.88	2.25
E-60	−85.6	7.74	13.7	4.7	6.95	2.25	1.06	5.77	7.00	1.00	0.75
E-84	−79.8	7.70	11.7	2.9	6.90	2.38	1.08	5.79	6.60	0.63	1.25
E-108	−76.3	7.82	9.8	2.4	6.94	1.88	1.13	4.83	6.39	0.75	2.50
E-132	−72.5	7.95	5.0	1.5	6.34	2.25	1.18	4.96	5.53	0.88	2.00
E-156	−77.4	7.93	5.0	1.3	6.56	2.00	1.18	3.12	5.48	0.63	2.00
E-180	−68.5	7.99	4.3	1.5	6.07	1.50	1.22	1.46	3.43	0.63	1.75

## Data Availability

This study includes all supporting data, which can be obtained from the corresponding authors upon request.

## Supplementary Materials

The following supporting information can be downloaded at https://www.mdpi.com/article/10.3390/toxics10120738/s1, Table S1: Characteristics of complete pore water chemistry results during compaction in different compaction rates. Table S2: Characteristics of complete pore water chemistry results during compaction in different compaction patterns.
