# Supplementary material for: Impact of Pressure on Arsenic Released from Pore Water in Clayey Sediment
Source: Toxics. 2022 Nov 29;10(12):738. doi: 10.3390/toxics10120738 (PMC9785127; doi:10.3390/toxics10120738)
Supplement: Supplementary file 1 [file toxics-10-00738-s001.zip › toxics-1997158-supplementary.pdf]

Supporting Information

Table S1. Characteristics of complete pore water chemistry results during compaction in different compaction rates.

Sample	Eh	pH	EC	Na	Mg	K	Ca	Zn	Al	Fe	Mn	Cl-	NO_3^-	SO_4^{2-}	As	As(Ш)	DOC	Fe^{2+}	NH_4-N
	mV		µs/cm	mg/L											µg/L	µg/L	mg/L	mg/L	mg/L
A-12	-20.5	7.9	636	2.80	4.51	0.170	23.4	0.003	0.077	1.88	1.11	4.60	7.64	7.83	10.1	2.6	6.93	1.38	0.50
A-24	-87.5	7.82	666	2.89	4.86	0.273	24.6	0.015	0.024	2.38	1.31	4.58	7.47	6.50	16.1	7.3	8.30	1.13	1.00
A-36	-93.4	7.64	682	4.13	5.26	0.205	26.2	0.008	0.055	3.00	1.43	4.52	6.18	6.25	12.3	5.1	6.82	1.00	0.75
A-48	-90.6	7.65	682	2.90	4.93	0.274	25.1	0.008	0.018	2.63	1.52	4.61	6.24	5.78	11.4	3.6	6.30	1.00	0.88
A-60	-92.0	7.62	683	3.02	5.01	0.127	25.3	0.008	0.018	2.75	1.60	4.46	5.55	5.52	13.4	4.2	6.25	1.00	1.25
A-72	-92.7	7.73	676	3.03	4.98	0.123	24.7	0.016	0.019	3.25	1.62	4.35	5.88	5.87	13.9	3.6	6.17	1.00	1.50

A-84	-80.6	7.84	672	3.09	4.95	0.139	25.2	0.006	0.03	2.25	1.63	4.37	5.35	5.44	12.6	3.7	6.45	0.75	1.25
A-96	-91.3	7.97	673	3.45	5.15	0.117	25.4	0.007	0.062	2.75	1.67	4.48	4.93	5.00	10.9	2.9	6.37	0.75	1.00
A-108	-84.5	7.95	667	2.86	5.04	0.226	24.6	0.009	0.023	2.63	1.65	4.28	3.70	4.99	8.7	2.2	6.41	1.00	1.25
A-120	-66.3	8.01	615	2.78	4.75	0.133	24	0.004	0.022	2.13	1.64	4.10	2.46	4.87	7.6	2.0	6.58	1.00	1.25
B-12	-15.7	8.09	794	3.17	6.07	0.323	27.3	0.010	0.034	5.50	0.97	4.21	7.30	7.09	14.1	3.9	6.01	2.44	2.50
B-36	-73.2	7.74	832	2.9	6.27	0.223	27.4	0.030	0.078	5.51	1.05	4.05	5.86	6.11	26.9	11.9	6.88	2.44	1.75
B-60	-89.3	7.18	777	2.99	6.91	0.212	30.4	0.010	0.118	7.00	1.16	3.98	3.73	5.27	36.4	22.0	7.54	2.63	2.75
B-84	-91.1	7.47	851	3.04	6.79	0.571	29.8	0.009	0.133	7.50	1.15	4.03	2.61	5.54	30.0	17.3	6.98	3.19	2.25
B-108	-101.9	7.61	851	3.01	6.79	0.200	30.0	0.012	0.030	4.28	1.15	4.02	1.83	5.25	24.2	12.1	6.79	2.25	0.75
B-132	-91.6	7.59	844	2.95	6.64	0.222	30.0	0.02	0.041	6.25	1.13	4.04	1.66	5.20	22.7	12.3	6.22	2.81	1.25
B-156	-97.2	7.68	844	3.73	7.06	0.220	31.3	0.012	0.050	5.40	1.12	4.23	1.08	4.96	20.3	6.4	5.74	1.31	2.50
B-180	-97.9	7.89	840	3.37	6.86	0.178	30.8	0.006	0.040	6.00	1.11	3.84	0.57	3.56	23.9	7.1	5.87	1.31	2.00
B-204	-86.0	7.95	839	3.78	6.96	0.233	30.8	0.008	0.022	5.75	1.11	4.01	0.56	3.93	14.9	4.0	5.60	1.88	2.00
B-228	-97.4	7.98	842	3.72	6.69	0.466	29.8	0.006	0.022	4.28	1.09	4.14	0.32	3.03	11.1	3.3	5.75	1.69	1.75
C-12	-25.6	7.84	849	2.89	6.43	0.192	30.6	0.017	0.043	5.38	1.17	4.48	7.92	7.52	15.7	4.7	6.22	2.25	2.00
C-36	-91.6	7.87	860	5.25	8.53	0.262	38.8	0.019	0.308	6.63	1.14	4.01	6.48	6.16	28.4	11.6	6.79	4.00	1.25

C-60	-87.4	7.12	796	3.93	6.94	0.182	32.4	0.020	0.089	7.50	1.23	3.89	5.84	5.92	30.5	17.4	6.81	2.50	2.00
C-84	-102	7.52	843	4.49	7.07	0.199	32.7	0.007	0.077	4.88	1.23	3.89	2.48	5.23	23.4	12.2	6.74	2.00	1.75
C-108	-97.2	7.65	850	3.23	6.67	0.173	31.8	0.018	0.031	7.00	1.20	3.80	1.66	5.36	24.8	13.1	6.29	2.00	1.00
C-132	-95.8	7.65	835	3.74	6.63	0.187	31.3	0.005	0.033	6.38	1.18	3.80	0.65	5.29	19.6	10.4	6.27	3.50	2.00
C-156	-96.4	7.64	845	3.03	6.53	0.143	30.3	0.011	0.090	6.75	1.17	4.59	0.57	4.90	19.4	7.8	6.05	2.00	2.25
C-204	-98.9	7.79	842	3.29	6.73	0.175	31.4	0.008	0.099	6.13	1.16	3.97	0.55	3.94	21.1	8.0	6.18	1.50	1.25
C-252	-107.8	7.84	836	3.69	6.5	0.475	30.4	0.008	0.037	5.13	1.12	3.80	0.58	3.79	15.7	5.0	5.99	1.75	1.25
C-300	-86.8	8.09	834	1.53	3.27	0.103	15.9	0.012	0.055	5.13	0.55	3.88	0.60	3.89	16.5	5.1	6.02	0.75	1.50
C-348	-92.5	8.23	815	1.51	3.29	0.117	15.6	0.013	0.054	2.00	0.55	2.24	0.50	3.42	13.2	3.6	6.16	1.00	1.50
C-396	-89.0	8.00	762	1.61	3.28	0.076	14.5	0.009	0.015	3.88	0.44	2.13	0.58	3.23	10.2	2.6	5.43	1.25	1.50
C-444	-76.6	7.89	764	1.54	3.06	0.245	13.6	0.011	0.029	2.30	0.41	2.88	0.52	3.52	6.7	1.3	5.52	1.60	1.25

Table S2 Characteristics of complete pore water chemistry results during compaction in different compaction patterns.

Sample	Eh	pH	Na	Mg	K	Ca	Zn	Al	Fe	Mn	Cl-	NO_3^-	SO_4^{2-}	As	As(Ⅲ)	DOC	Fe^{2+}	NH_4-N
	mV	mg/L												μg/L	μg/L	mg/L	mg/L	mg/L
D-12	-18.9	7.94	2.91	5.48	0.321	20.3	0.055	0.127	5.25	0.88	4.30	7.21	7.95	16.8	4.4	5.97	2.25	0.50
D-36	-59.9	7.74	2.66	4.06	0.128	20.7	0.009	0.022	5.75	0.88	4.19	5.67	6.22	23.9	11.5	6.79	2.00	0.75
D-60	-94.0	7.23	2.41	4.02	0.171	20.2	0.006	0.015	6.50	0.93	4.09	3.04	6.02	32.1	17.2	6.90	1.50	1.25
D-84	-108.9	7.58	2.75	4.25	0.137	21.4	0.018	0.043	5.25	1.00	4.14	2.24	5.81	27.4	15.3	5.85	1.50	1.25
D-108	-97.5	7.53	4.08	4.45	0.159	22.4	0.008	0.125	4.75	1.02	4.02	1.46	5.76	27.9	13.4	5.71	2.25	1.25
D-120	-95.0	7.61	2.55	4.26	1.910	21.1	0.001	0.002	4.00	0.78	7.29	0.57	5.79	42.9	25.8	4.49	2.50	1.25
D-132	-91.4	7.71	1.29	3.40	1.680	10.8	0.018	0.067	3.75	0.59	3.99	0.51	5.33	47.4	21.5	4.99	1.75	1.00
D-144	-96.6	7.75	3.61	4.12	0.359	20.7	0.013	0.038	5.75	1.10	4.35	0.54	4.51	34.9	11.0	3.32	1.50	1.00

D-156	-88.9	7.73	2.48	4.08	1.780	20.2	0.009	0.020	4.50	1.09	4.01	0.58	4.75	37.0	11.3	2.21	2.75	1.25
D-168	-90.5	7.77	2.54	4.45	0.210	21.3	0.024	0.05	5.50	1.08	4.19	0.52	3.23	33.4	8.4	1.71	2.00	1.00
E-12	-18.1	7.91	3.28	4.13	0.255	20.2	0.010	0.039	2.00	0.91	4.39	8.21	7.12	10.8	2.9	6.67	1.25	2.50
E-24	-62.3	7.83	1.35	1.85	0.175	9.24	0.013	0.015	2.00	0.46	2.32	7.88	7.07	14.4	6.8	8.92	0.75	1.75
E-36	-75.0	7.67	2.35	3.86	0.184	19.5	0.025	0.031	2.00	0.98	4.32	5.91	6.83	12.8	5.4	6.95	1.00	2.75
E-48	-73.9	7.70	4.33	5.23	0.282	25.6	0.008	0.156	2.63	1.05	4.14	5.45	6.98	12.7	4.6	6.90	0.88	2.25
E-60	-85.6	7.74	3.82	3.98	1.370	21.7	0.012	0.043	2.25	1.06	7.88	5.77	7.00	13.7	4.7	6.95	1.00	0.75
E-84	-79.8	7.70	2.30	3.86	0.187	19.3	0.005	0.021	2.38	1.08	4.11	5.79	6.60	11.7	2.9	6.90	0.63	1.25
E-108	-76.3	7.82	2.36	3.96	0.168	19.3	0.012	0.038	1.88	1.13	4.37	4.83	6.39	9.8	2.4	6.94	0.75	2.50
E-132	-72.5	7.95	4.37	4.61	0.226	22.3	0.016	0.084	2.25	1.18	4.14	4.96	5.53	5.0	1.5	6.34	0.88	2.00
E-156	-77.4	7.93	2.41	3.99	0.167	19.5	0.008	0.028	2.00	1.18	4.16	3.12	5.48	5.0	1.3	6.56	0.63	2.00
E-180	-68.5	7.99	2.50	4.10	0.146	19.8	0.008	0.044	1.50	1.22	4.12	1.46	3.43	4.3	1.5	6.07	0.63	1.75
